# Case of Acute Gastritis

**Published:** 1865-03

**Authors:** Hiram Nance

**Affiliations:** Kewanee, Illinois


ARTICLE IX.

### CASE OF ACUTE GASTRITIS.—SUDDEN DEATH.

BY HIRAM NANCE, M.D., Kewanee, Ill.

I was called on Friday evening, March 3d, at twilight to see Miss Rosanna Wiley, aged 17. Found her sitting up in a chair Pulse about natural; tongue slightly coated; countenance rather depressed; temperament nervo-bilious. She complained of some pain and oppression in the epigastric region, extending to the right hypochondriac region, and, slightly, to the left hypochondriac region. I saw nothing in the case, arousing my suspicions that anything very serious was pending. I diagnosed hæpatic torpor, or slight congestion of the stomach and liver. In fact, I regarded the case as one of our ordinary bilious attacks of this climate, and founded my treatment upon this basis. She had been to school the day previous, and, in fact, had not as yet taken her bed. I made inquiries, and found that her appetite had been failing for some eight or ten days, and she had been complaining for this length of time with slight pain and oppression in the parts previously spoken of. I ordered her three powders, each composed of prot. ch. hyd. grs. v, rhei. rad. pulv. grs. xv, one to be taken every alternate or third night, if she did not seem much improved on the first, and requested some one of the family to call the next day at my office, and get some vegetable bitter in wine, to be repeated morning, noon, and night. The latter was not called for; but on the next evening (Saturday), about the same time of day, I was again summoned to see her. Dr. J. F. TODD, a former student of mine, but now a practitioner of medicine, residing and doing business at Wyanet, Bureau Co., happened to be at my house. I invited him to accompany me to see the patient, not that I regarded the case a perplexing and difficult one, but through courtesy. We found her in bed, suffering from pain in the stomach; complained of some burning sensation and oppression as formerly; countenance rather pallid, though not more than is usually seen in diarrhœa, or other common bowel affections in their formative stages; pulse some accelerated, amounting to about 100 or 110, rather small and quick; tongue moist, and slightly furred, ordinary color; no elevation of the papilla; very little sickness at the stomach. I conversed with Dr. TODD, and we came to the conclusion, as the bowels had moved but little from the powder I had ordered the night previous, that we continue the same medicine until free evacuations were obtained. I ordered the same that had been left the previous evening (cal. and rhei.), and if it did not move by morning, to give the second dose. We conversed about the case when we left the house, and I gave it as my opinion that gastritis might be the result, founding my opinion upon the oppression about the stomach; some pain in the epigastric region; small pulse; thirst, sickness, and burning sensation, though the latter symptom not very well marked. I omitted to mention that I also left four powders, each containing grs. ij prot. ch. hyd. and $\frac{1}{6}$ gr. acetate morphia. Those were to be given every two, three, or four hours after the operation of the physic, if the pain and uneasiness continued. I had not been at home more than two hours, before a messenger came after me, saying she was suffering much pain. I told him I could do nothing more, as I had just left the house, and, instead of waiting until next morning to give the second dose of medicine to move the bowels, to repeat it in four hours, and, then, if the pain continued, to give the anodyne and alterative powders I had left until she was relieved. I also ordered sinapisms and hop poultices, and to abstain, as much as possible, from drinks.

Next morning (Sunday), called to see her, about 10 A.M. Found her raised up in bed, with a lady supporting her in a sitting position. Pulse from 120 to 130, very weak; face pallid and waxy; lips purplish; tongue moist, slightly furred; great depression; continued nausea, and occasional vomiting; excessive restlessness; extremities cool; surface of body natural; skin but very little moist. Those symptoms alarmed me very much, in fact, I regarded her in a state of semi-collapse, from which I feared she would never react. I immediately ordered her one spoonful of brandy and ¼ gr. of acetate of morphia, sinapisms to be applied to the stomach, bowels, and extremities. I also, immediately, ordered a messenger to go for Dr. TODD, who was only a few block's distant, and in ten minutes time he was with me. I expressed my fears that she would never react. The irritation of the stomach was so great that nothing could be retained. We gave her morphia, sul. æther, Hoff's anodyne, brandy, ice, small doses of calomel, &c., &c., all to no purpose. Withheld drinks, giving only small pieces of ice in lemonade, but nothing proved of any avail. We remained with her until 2 P.M., then left for half an hour. I then called and staid an hour; went away, and returned in two hours. When I returned, I found her still sinking; vomiting had ceased, but the nausea still continuing; pulse a mere flutter.

I requested Dr. SCOTT, of this place, to call in with me and see her. He came in about 8½ P.M. Pulse at one wrist had then ceased, and very faint at the other. Half an hour longer she was pulseless; *intellect perfect.* I informed her she could not live but a very short time. She seemed astonished; said she felt quite easy. She called her friends around her, bid them affectionate farewells, and in half an hour death closed the scene.

I report this case for its peculiarity, for its sudden termination in collapse and death. No *post mortem* was obtained. Now, the question arises, was it a genuine case of gastritis? Dr. TWEEDIE, in his work on Practical Medicine, says, "if the disease (acute gastritis) does not *terminate in death in a few hours*, or by the second or third day, it may extend to two or three weeks, and still prove mortal; or it may pass into chronic gastritis of indefinite duration." He further says, "death in rapid cases is produced by depression of the vital functions." Acute gastritis is usually produced from poisons, concentrated acids, or cold drinks when the person is over heated. None of these could I attribute this attack to. She had been complaining, as I observed, several days with oppression, &c., of the stomach, before the urgent symptoms made their appearance. She apparently, and not only apparently, but did actually die from the *shock* the nervous system received before reaction took place.

The great solar plexus of nerves composed of ganglions and filaments intervening and anastimosing with each other from the great sympathetic, also branches from the pneumo-gastric or eighth pair, is minutely distributed upon this great plexus. This great solar plexus of nerves is situated in the spinal column posterior to the stomach, liver, and spleen, and gives off inferior plexus to the diaphragm, liver, and stomach, also the superior and inferior mesentric. The kidneys are likewise supplied with nerves from this plexus. Thus we see this nervous plexus is of great magnitude, and if it should receive a severe shock from any source, either idiopathic or from injury externally applied, death might speedily result. Now, my opinion is, that the great lesion was about this great plexus of nerves. That there was some congestion of the liver, spleen, and stomach, I do not doubt; but I should doubt extremely if inflammatory symptoms had as yet set up in the stomach when death took place, notwithstanding TWEEDIE says death may take place in a few hours. You will observe there was not a flushed countenance, neither was there a red tongue, nor any great degree of burning in the epigastrum. If in acute gastritis we must have those symptoms, then inflammatory symptoms had not set up, and she died from the collapse of the nervous symptoms produced by inflammation of the stomach. Had she reacted, in my opinion, active well-marked gastritis would have been the result.

In conclusion, I might say, that my treatment was not such as is ordinarily pursued in cases of acute gastritis, neither do I think any *sane* physician would have adopted such treatment under such circumstances. Who wouldn't give brandy and other stimulants to a patient in a dying condition? And where would you find a physician fool enough in our profession to bleed, cup, leech, and use other antiphlogistics when the last lingering spark was fast fading away?

I omitted to say in the proper place, that I diligently enquired if my patient had either ate or drank any unusual diet recently, and found that on Thursday she had been eating oysters with the rest of the family. Had others of the family been ill, I might have attributed the exciting cause to the oysters, but they all remained well.

